# Motor Learning in a Complex Motor Task Is Unaffected by Three Consecutive Days of Transcranial Alternating Current Stimulation

**DOI:** 10.3390/bioengineering11080744

**Published:** 2024-07-23

**Authors:** Erik W. Wilkins, Milan Pantovic, Kevin J. Noorda, Mario I. Premyanov, Rhett Boss, Ryder Davidson, Taylor A. Hagans, Zachary A. Riley, Brach Poston

**Affiliations:** 1 Department of Kinesiology and Nutrition Sciences, University of Nevada-Las Vegas, Las Vegas, NV 89154, USA; wilkie1@unlv.nevada.edu; 2 Interdisciplinary Ph.D. Program in Neuroscience, University of Nevada-Las Vegas, Las Vegas, NV 89154, USA; 3 Health and Human Performance Department, Utah Tech University, St. George, UT 84770, USA; milan.pantovic@utahtech.edu; 4 School of Medicine, University of Nevada-Las Vegas, Las Vegas, NV 89154, USA; noordk1@unlv.nevada.edu (K.J.N.); premayno@unlv.nevada.edu (M.I.P.); bossr1@unlv.nevada.edu (R.B.); davidr6@unlv.nevada.edu (R.D.); hagant1@unlv.nevada.edu (T.A.H.); 5 Department of Kinesiology, Indiana University Purdue University Indianapolis, Indianapolis, IN 46202, USA; zariley@iupui.edu

**Keywords:** transcranial alternating current stimulation, transcranial direct current stimulation, motor learning, motor skill, transcranial magnetic stimulation, motor evoked potential

## Abstract

Transcranial alternating current stimulation (tACS) delivered to the primary motor cortex (M1) can increase cortical excitability, entrain neuronal firing patterns, and increase motor skill acquisition in simple motor tasks. The primary aim of this study was to assess the impact of tACS applied to M1 over three consecutive days of practice on the motor learning of a challenging overhand throwing task in young adults. The secondary aim was to examine the influence of tACS on M1 excitability. This study implemented a double-blind, randomized, SHAM-controlled, between-subjects experimental design. A total of 24 healthy young adults were divided into tACS and SHAM groups and performed three identical experimental sessions that comprised blocks of overhand throwing trials of the right dominant arm concurrent with application of tACS to the left M1. Performance in the overhand throwing task was quantified as the endpoint error. Motor evoked potentials (MEPs) were assessed in the right first dorsal interosseus (FDI) muscle with transcranial magnetic stimulation (TMS) to quantify changes in M1 excitability. Endpoint error was significantly decreased in the post-tests compared with the pre-tests when averaged over the three days of practice (*p* = 0.046), but this decrease was not statistically significant between the tACS and SHAM groups (*p* = 0.474). MEP amplitudes increased from the pre-tests to the post-tests (*p* = 0.003), but these increases were also not different between groups (*p* = 0.409). Overall, the main findings indicated that tACS applied to M1 over multiple days does not enhance motor learning in a complex task to a greater degree than practice alone (SHAM).

## 1. Introduction

Motor learning refers to the relatively permanent improvement in a motor skill through deliberate practice over time [1,2]. Accordingly, the process of motor learning is characterized by the development of the ability to perform a motor skill with increasing levels of precision, movement efficiency, and automaticity [1]. To optimize motor learning, it is not enough to be taught correctly how to perform a motor skill, extensive practice and ideal practice strategies are also needed. Since motor practice is the primary factor responsible for motor learning, substantial research efforts have been made over many decades to determine the best parameters of motor practice to induce motor learning [3]. While these studies have been valuable, the vast majority involved simple motor tasks that were completely novel to the participants and were conducted in laboratory conditions. Therefore, it is not surprising that subsequent research found that many of the practice principles developed in studies involving simple motor tasks are not generalizable to the motor learning [3] and control processes [4,5] of complex motor tasks. Although many advances have been made in the methods and physiological mechanisms underlying improving motor learning through practice, a few modalities exist that can enhance motor learning to a greater degree than progressive, well-designed practice regimes alone. This is particularly evident in healthy young adults with difficult motor tasks, where ceiling effects often impose an upper limit on motor skill development over both short and relatively longer time periods. Therefore, the development of interventions that can improve motor learning beyond the levels that can be achieved by intensive practice alone would have substantial biomedical significance and practical applications.

Recently, non-invasive brain stimulation methods have been developed that have the potential to enhance motor skill and learning in a variety of populations, including older adults [6,7], individuals with motor disorders [8,9,10], and healthy young adults [11]. Transcranial direct current stimulation (tDCS) has been the most widely studied non-invasive brain stimulation method and the most practical to implement in both research and real-world applications [12]. tDCS involves passing a weak electric current over a targeted brain region [13], most commonly the primary motor cortex (M1), with the goals of increasing cortical excitability [14], motor skill [15,16,17], and motor learning [11,18,19,20,21,22]. Numerous acute studies involving fine control of the hand and arm have demonstrated an increase in motor skill of about 10–15% after one 20-min session of M1-tDCS [11,22]. Repeated M1-tDCS application over 3–5 days can sometimes increase performance in young adults by approximately 30–40% beyond practice alone in relatively simple motor tasks such as isometric pinch grip tasks [18,19,20]. Nonetheless, there may be further opportunities for improvement by modification of the tDCS parameters (e.g., current strength, duration, electrode montage, stimulation site, number of stimulation sites, and timing relative to practice) or by using other methods of non-invasive brain stimulation that are variations of tDCS.

Transcranial alternating current stimulation (tACS) has recently emerged as perhaps the most promising tDCS variation [23,24,25,26,27,28,29]. Although much less research has been performed on tACS compared to tDCS, the number of studies published on the technique is increasing rapidly [30]. Most importantly, some initial studies have shown increases in motor skill acquisition similar in magnitude to those achieved with tDCS. For example, Sugata and colleagues (2018) [24] reported that a single session of 70 Hz tACS applied to M1 substantially increased motor skill learning in a 12-digit motor sequence button pressing task of the right hand compared to SHAM stimulation. In addition, these behavioral improvements were accompanied by increased power in the beta-band, as measured by magnetoencephalography (MEG). Similarly, a series of studies by Miyaguchi and colleagues [31,32,33,34] demonstrated increases in motor skill learning, although in these studies, tACS was applied to M1 and the cerebellum (c-tACS) simultaneously. tACS can also increase M1 excitability [23,35,36], although perhaps not to the same magnitude as tDCS. However, tACS has a potential advantage over tDCS due to its ability to induce the entrainment of large groups of cortical neurons at specified frequencies [23,37,38]. This can be accomplished both within a given targeted brain area and between two functionally and anatomically linked brain areas [24,29,37,38]. However, similar to the preponderance of tDCS studies, most tACS-related motor skill research has involved a single stimulation and practice session of relatively simple hand and arm tasks. Furthermore, these studies have not measured changes in M1 excitability and motor skill in the same study. Therefore, it is unknown if tACS can increase motor learning in a complex, multi-joint task involving whole body control and endpoint accuracy, especially in healthy young adults who may have less room for performance improvements compared to older adults or patients with movement disorders.

The primary aim of this study was to assess the impact of tACS applied to M1 over three consecutive days of practice on the motor learning of a challenging overhand throwing task in young adults. The secondary aim was to examine the influence of tACS on M1 excitability. Based on a series of studies involving M1-tACS [24] and dual-site M1-tACS and c-tACS [31,32,33,34] in simple motor tasks, it was predicted that tACS application would induce greater enhancements in motor learning in the overhand throwing task compared to SHAM stimulation. Similarly, it was hypothesized that tACS would also increase M1 excitability [23,35,36], whereas SHAM stimulation would have no effect. Relatedly, if significant effects of tACS on motor skill learning were to occur relative to SHAM stimulation, it was expected that increases in motor learning would be positively correlated with increases in M1 excitability, as has been shown in a few early tDCS studies [39,40]. The overhand throwing task and combination of other task parameters (e.g., small target size and long throwing distance) employed were selected within laboratory space constraints to present a notably complex model motor task to participants for the evaluation of motor learning.

## 2. Materials and Methods

### 2.1. Participants

A total of 24 healthy young adults participated in this study (20 males and 4 females; mean age: 24.9 ± 3.4; with 10 men and 2 women in each group). The participants were recruited through the use of recruitment flyers posted in university buildings. All study participants exhibited right-arm dominance, as indicated by their Edinburgh Handedness Inventory [41] laterality quotient scores. Comprehensive screening ensured the absence of any neurological or psychiatric pathologies, uncontrolled medical comorbidities, or contravention of international transcranial magnetic stimulation (TMS) exclusion criteria [42]. Individuals who were currently actively engaged in recreational, collegiate, or professional throwing disciplines were also ineligible for study inclusion. Prior to enrollment, subjects provided written consent following a detailed explanation of this study’s objectives and procedures. While participants possessed awareness of this study’s overarching goals, they remained blinded to the specific treatment condition assigned to them throughout the experimental process. All experimental procedures were consistent with the ethical guidelines stipulated in the Declaration of Helsinki. Approval for this study was obtained from the Institutional Review Board at the University of Nevada, Las Vegas.

The sample size was estimated using G*Power 3.1.9.7 software based on the data and effect size (η_p_^2^ *=* 0.188) from Sugata and colleagues (2018) [24]. A priori power analysis for an independent *t*-test using a two-tailed hypothesis test, a significance level of α = 0.05, and a power of 0.8 resulted in a sample size estimate of 22 participants (11 per group). Finally, we settled on a sample size of 24 (12 per group), as we predicted an attrition rate of at most 10% based on our previous studies in young adults.

### 2.2. Experimental Design and Protocol

**Figure 1:**
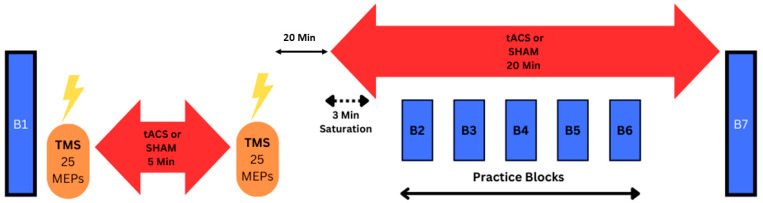
Schematic of the experimental protocol of this study. A total of three identical experimental sessions were completed on three consecutive days. A depiction of the one experimental session is shown for illustration. The experimental protocol included a pre-test block of overhand throwing trials, a TMS testing protocol of M1 excitability changes in response to five minutes of tACS, a 20-min break, five practice blocks of overhand throwing trials performed in association with concurrent tACS application for 20 min, and a post-test block of overhand throwing trials.

This study implemented a double-blind, SHAM-controlled, randomized, between-subjects experimental design. Overall, the design and methodology were nearly identical to two prior studies performed in our laboratory, with the exception that those studies involved tDCS applied to M1 [21] and the cerebellum (c-tDCS) [43], respectively. Participant assignment to either the tACS or SHAM stimulation group was carried out using Research Randomizer (www.randomizer.org accessed on 17 June 2024) by an investigator not involved in data collection [44,45]. The SHAM condition served as the control or placebo, utilizing established SHAM procedures from previous tDCS and tACS studies. Participants completed three consecutive experimental sessions, each lasting approximately two hours, at consistent times of day throughout the entirety of this study. Other than a brief familiarization phase at the start of the first session involving didactic video instruction and throwing demonstrations by investigators, all sessions were identical except for the type of stimulation administered (tACS and SHAM) to each of the two groups. However, the participants themselves did not engage in any familiarization or practice throwing trials before the experimental procedures. Thus, total naivety in this particular overhand throwing task and experimental environment was ensured prior to experimentation. In general, the experimental sessions encompassed the following sequence of steps: (1) a pre-test block of overhand throwing trials, (2) TMS testing of tACS effects on M1 excitability, (3) practice blocks of overhand throwing trials concurrent with stimulation, and (4) a post-test block of overhand throwing trials. These steps are described in greater detail in subsequent sections, and a schematic of the experimental protocol is provided in Figure 1. Throughout all experimental conditions, investigators conducting the experiments remained blind to participants’ group assignments. Specifically, the investigator responsible for operating the tACS device and applying stimulation was not involved in other experimental procedures [44,45].

### 2.3. Experimental Procedures

#### 2.3.1. Pre-Test Block

A pre-test block comprising 10 overhand throwing trials was conducted to establish the baseline performance levels for both groups on Day 1, prior to any stimulation application. Similarly, the preliminary evaluations on Days 2–3 followed an identical protocol, serving as baselines uninfluenced by stimulation but potentially affected by consolidation effects from the preceding day. The selection of 10 trials per block for all preliminary evaluations was based on prior research [21,43,46], ensuring adequate baseline data without overly impacting subsequent practice block performance curves. Furthermore, this maintained consistency with the number of trials per block in the practice and post-test phases of the experiments. Finally, the absence of concurrent tACS during the pre-test block facilitated the assessment of the unique contributions of online and offline learning to overall motor learning when combined with the results of the post-test block (refer to Section 2.7 Statistical Analysis).

#### 2.3.2. TMS Measurements of M1 Excitability

A Magstim 200^2^ TMS device (The Magstim Company Ltd., Spring Gardens Whitland, Carmarthenshire, UK) connected to a double 70 mm remote control figure-of-eight coil was used to perform single-pulse TMS. The coil was positioned tangentially to the scalp, its handle directed backwards and laterally at a 45-degree angle from the midline, ensuring stimulation over the “motor hot spot” of the first dorsal interosseous (FDI) muscle of the left M1, thereby evoking MEPs in the contralateral FDI muscle of the right hand. The MEP amplitude served as an index of net M1 excitability. EMG activity of the FDI muscle was recorded using surface electrodes arranged in a belly tendon montage, and data were acquired and recorded using Cambridge Electronic Design (CED; Cambridge, UK) hardware (1902 amplifiers, micro 1401 data acquisition interface) and software (Signal 5.04). Participants were positioned with their forearm on a table, wrist in a neutral position, hand prone, elbow flexed to ~90 degrees, and shoulder abducted to ~45 degrees, maintaining consistent posture across recordings [21,43] to minimize any influences of changing arm position on MEP amplitudes [47]. Prior to the TMS blocks, participants were provided with instructions on using the visual EMG feedback [48] given on a monitor placed in front of them to assure that the right FDI remained at rest for all TMS testing. Continuous monitoring by one investigator further ensured the FDI muscle remained at rest during all recordings. Accordingly, this investigator gave participants verbal feedback, if warranted, if the FDI EMG signal indicated muscle contraction at any time point during the TMS testing.

The TMS testing procedures were the same as described in detail in prior tDCS studies [21,43] and entailed several sequential steps: (1) the FDI hot spot was identified by administering suprathreshold TMS pulses to optimize coil position until the scalp area corresponding to the FDI motor hot spot was identified and marked on a scalp cap [49]; (2) the 1 mV stimulation as a percentage of maximum stimulator output (%MSO) was determined using the set of methods developed in two previous studies [21,43]. In brief, a series of TMS pulses were given at a moderate stimulation intensity, which was adjusted while MEPs were monitored online in the Signal software. Once MEP amplitudes were as close as reasonably possible to 1 mV on average, the software was reset; (3) the Pre-TMS test block was performed and 25 MEPs were collected with the previously determined 1 mV stimulation intensity; (4) 5 min of tACS or SHAM stimulation was applied to the left M1; (5) the post-test TMS block was completed as soon as stimulation ended using the same TMS stimulation intensity as before; and (6) a 20-min rest period (inter-stimulation period) was enforced, ensuring a delay between the end of the 5-min tACS application and the subsequent beginning of the 20-min period and associated overhand throwing practice blocks.

This complex paradigm, involving a 5-min tACS application followed by a 20-min break, was devised to address methodological concerns surrounding MEP measurements before and after tDCS. Informed by prior research focused on the effects of different tDCS duration protocols on M1 excitability, the paradigm underwent extensive piloting in the laboratory for the prior companion studies utilizing M1 and c-tDCS [21,43]. The paradigm was originally developed with the aim of reconciling three methodological considerations: (1) tDCS applied for 3–5 min induces MEP amplitude increases lasting 3–5 min post-stimulation [50,51,52]; (2) a 20–30-min break between tDCS applications sustains MEP increases, while shorter breaks result in inhibition [50,51]; and (3) tDCS-induced MEP increases can be nullified by muscle contractions, movement, and related activities, potentially rendering post-practice MEP measurement meaningless [53,54,55,56,57]. Thus, the current paradigm was designed to mitigate this limitation while enabling measurement of potential associations between MEP increases and motor learning enhancements [39,40,58], under the critical assumption that the second tACS application had equivalent effects on M1 excitability as the first, as shown in tDCS studies [50,51]. Overall, this methodology appeared to work as intended in our aforementioned prior studies [21,43].

#### 2.3.3. Practice Blocks

The practice blocks were conducted simultaneously with either tACS or SHAM stimulation, encompassing a total practice and stimulation period of 20 min (Figure 1). The practice blocks segment of this study unfolded in several stages. First, the stimulator was activated for 3 min, while subjects stood quietly before initiating the first block of overhand throwing trials [21,43,46]. Second, a sequence of 5 blocks of overhand throwing trials ensued, with each block consisting of 10 overhand throws. These blocks were executed within the remaining 17 min of stimulation, with each block requiring approximately 1 to 1.5 min to complete, and a 2-min rest interval was incorporated between blocks. These block parameters and duty cycle of throws were selected in an attempt to optimize the trade-off between performing as many trials as possible while minimizing the potential negative influences of muscle fatigue on muscle activation patterns [59] and motor learning [60]. Finally, the stimulator remained active after the conclusion of the last block of overhand throws, typically lasting 1–2 additional minutes to fulfill the 20-min stimulation period.

#### 2.3.4. Post-Test Block

Following the culmination of the practice blocks and the 20-min stimulation period, participants maintained a stationary stance, while the inactive electrode montage persisted on the head. They observed a 5-min rest interval before engaging in the post-test block, comprising 10 trials. As alluded to above, the execution of the post-test blocks without concurrent tACS facilitated the calculation of both online and offline learning effects on total motor learning (refer to Section 2.7 Statistical Analysis).

### 2.4. tACS and SHAM Stimulation

A NeuroConn DC Stimulator Plus/MR device (Neurocare Group AG, NeuroConn GmbH, Ilmenau, Germany) was employed for the administration of tACS of M1, delivering a current intensity of 1 mA at 70 Hz [24] through a pair of 5 × 7 cm rubber electrodes ensconced within saline-soaked sponges. The anode was positioned directly over the FDI motor “hotspot” of the left M1, while the cathode was situated over the contralateral right supraorbital area, which is typically referred to as the M1-SO electrode montage. Securing the anode and cathode in place involved separate rubber elastic straps. As previously noted, tACS was applied for 5 min between the TMS pre-test and post-test blocks, as well as for 20 min during the overhand throwing practice blocks, utilizing identical stimulation parameters (70 Hz and 1 mA current strength). During overhand throwing trials, the stimulation device was housed within a small backpack [21,43,46], whereas during MEP testing, the stimulator was positioned behind the participant on a table. While alternative tACS parameters exist that may enhance motor learning, the selected combination of tACS frequency, electrode montage, and current intensity was chosen based on the study of Sugata and colleagues (2018) [24]. SHAM stimulation adhered to the established protocol in the field [61], as has been implemented in previous studies [62,63].

### 2.5. Overhand Throwing Task

The overhand throwing task replicated procedures outlined in a previous single-session c-tDCS study [46] and our two previous three-day tDCS studies involving c-tDCS and M1-tDCS [21,43] that were executed with closely aligned experimental protocols. Participants positioned themselves behind a designated line on the floor, situated 6 m from a cement wall. Affixed to the wall was a securely fastened wooden board that displayed a laminated poster covered in clear tape. The poster depicted a large overall target area, but with a (1 cm diameter) “bull’s-eye” target at its center. Participants used their dominant right arm to throw a tennis ball in a manner similar to a baseball throw, aiming to achieve precision by targeting the “bull’s-eye” at the center of the target area. Each throw was followed by a visual feedback assessment of the ball’s endpoint relative to the target center. Participants were instructed to endeavor to minimize error distance on subsequent attempts. An investigator stationed near the participant coated the ball with red chalk both before and midway through each block of 10 trials, which marked the final endpoint position upon hitting the target. Subsequently, the same investigator retrieved the ball after a rebound and returned it to the participant for the next trial. Each mark, denoted by a trial-numbered circular sticker, was documented by a second investigator stationed near the target area. Following each trial block (interspersed with participant rest intervals), the sticker’s *x* and *y* endpoint coordinates were measured, recorded, and directly entered into an Excel file on a laptop by 2–3 investigators working synergistically on those tasks. Finally, the stickers were removed from the target area between trial blocks, and the process was repeated for subsequent blocks.

Throughout all trial blocks, the overhand throwing task remained consistent, with participants consistently wearing a snug-fitting backpack housing the tDCS device. Importantly, the tDCS device was activated solely during practice blocks (Figure 1). Therefore, it remained inactive during the pre- and post-test blocks of overhand throwing trials, but the inert electrode montage was kept on the participant’s head. The arrangement of the backpack, stimulator, and associated tACS electrode montage posed no hindrance to task performance [21,43,46], ensuring overhand throws were executed under identical experimental conditions and without spatial constraints. Collectively, the combination of the overhand throwing task, diminutive target size, and substantial throwing distance were selected within laboratory constraints to present a notably challenging motor skill. The unique physiological and biomechanical aspects of overhand throwing that justify it being referred to as a complex motor task have been detailed in depth in our prior publications [21] and in review articles [64,65]. Briefly, it is a three-dimensional, unconstrained multi-joint movement characterized by interaction torque regulation [66,67,68,69], precise finger force timing for endpoint control [70,71,72,73,74,75], and antagonistic muscle activation [69,70].

### 2.6. Data Analysis

The primary outcome measure was the endpoint error in the overhand throwing task, while the secondary outcome measure was the MEP amplitude. The endpoint error was determined following methodologies established and briefly described in prior research [21,43,49], while detailed procedures for endpoint error calculation in goal-directed tasks can be found in Poston and colleagues (2013) [76]. The Pythagorean theorem was used to calculate the shortest distance between the target center’s coordinates and the final coordinates of the ball’s endpoint. A custom Microsoft Excel program was used to compute the endpoint error for each trial based on the ball’s endpoint coordinates. The average endpoint error across the 10 overhand throwing trials within each trial block was considered the final endpoint error value for analysis. In contrast, MEP amplitude data were subjected to analysis using a custom script in Signal software (Cambridge Electronic Design, Cambridge, UK). MEP size was determined as the peak-to-peak amplitude for each MEP, and the average of the 25 MEPs within each TMS test block was used for analysis [77].

### 2.7. Statistical Analysis

All statistical analyses were conducted using IBM SPSS Version 28.0.1.0. The analysis of endpoint error followed a methodology primarily based on a three-day c-tDCS study by Cantarero and colleagues (2015) [78], while also being very similar to our previous studies [21,43,46]. Accordingly, the endpoint error analysis proceeded through three steps. First, the endpoint error obtained solely from the test blocks underwent a 2 group (tACS, SHAM) × 3 day (1, 2, 3) × 2 test (pre-test, post-test) three-way mixed ANOVA with the factor group being between-subjects and the factors day and test being within-subjects. This analysis used data only from test blocks since no stimulation was applied during these blocks, facilitating comparison with the results of Cantarero and colleagues (2015) [78] and those of other three-day tDCS studies by other researchers [18,19,20]. Second, all of the endpoint error data from each day (test and practice blocks) was analyzed with a two-way mixed ANOVA: 2 group (tACS, SHAM) × 3 day (1, 2, 3) with the factor group being between-subjects and the factor day being within-subjects. Accordingly, this analysis used the average endpoint error value across all seven blocks (2 test blocks and 5 practice blocks) performed each day to provide a comprehensive representation of overall daily performance variations, considering the task’s difficulty. Third, the online, offline, and total learning effects were compared between groups using a series of three separate unpaired two-tailed *t*-tests [78].

For MEP amplitude, the data underwent a 2 group (tACS, SHAM) × 3 day (1, 2, 3) × 2 test (pre-test and post-test) three-way mixed ANOVA with the factor group being between-subjects and the factors day and test being within-subjects. In addition, similar to two previous studies with the same design [21,43], it was planned to perform bivariate linear regression analyses to examine the relationship between changes in MEP amplitudes and changes in endpoint error if they were to occur [21,43]. However, ultimately, these analyses could not be performed in the present study because there were not enough participants in either group who demonstrated both an increase in MEP amplitude and an increase in endpoint accuracy (lower endpoint error) within any of the three days.

Bonferroni adjustments were applied for post hoc comparisons, where necessary, to identify significant differences. Effect sizes are reported as the partial eta squared for ANOVAs and Cohen’s *d* for the *t*-tests. The significance level was set at α < 0.05 for all analyses. Data are depicted as means ± standard errors in the figures, whereas means ± standard deviations are reported in descriptions in the text.

## 3. Results

### 3.1. Endpoint Error

**Figure 2:**
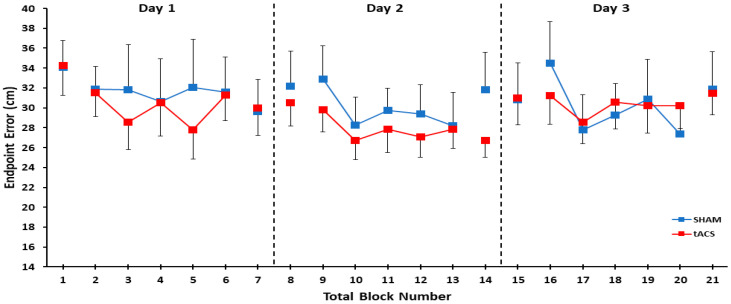
Endpoint error in the overhand throwing task for the tACS and SHAM groups as a function of block number for all three days of practice. Over the entire course of practice, there were no significant differences in endpoint error between the tACS and the SHAM groups *(p* = 0.741). Thus, participants only demonstrated small, non-significant improvements in throwing performance from the beginning to the end of practice (no total learning effect). However, endpoint error was significantly decreased in the post-test blocks compared with the pre-test blocks when averaged over all three days of practice (test main effect: *p* = 0.046; online effects), but this decrease was not significantly different between the tACS and SHAM groups (group main effect: *p* = 0.474).

The 2 group (tACS, SHAM) × 3 day (1, 2, 3) × 2 test (pre-test, post-test) three-way mixed ANOVA revealed that the main effect for group (*p* = 0.741, η_p_^2^ = 0.005) and main effect for day (*p* = 0.433, η_p_^2^ = 0.037) were both non-statistically significant. However, there was a significant main effect for the test (*p* = 0.046, η_p_^2^ = 0.169), which indicated that the endpoint error was significantly lower in the post-tests compared with the pre-tests when averaged over the three days of practice. In contrast, the day × group interaction (*p* = 0.307, η_p_^2^ = 0.052), test × group interaction (*p* = 0.474, η_p_^2^ = 0.024), day × test interaction (*p* = 0.307, η_p_^2^ = 0.052), and day × test × group interaction (*p* = 0.844, η_p_^2^ = 0.08) were all non-statistically significant (Figure 2).

**Figure 3:**
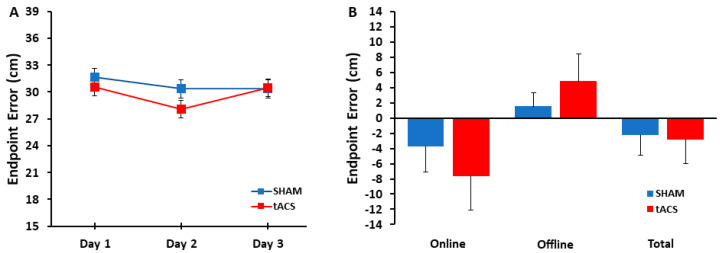
Endpoint error in the tACS and SHAM groups. (**A**) Endpoint error was calculated as the shortest absolute distance between the target center’s coordinates and the final coordinates of the ball’s endpoint. Endpoint error was not significantly different between the tACS and the SHAM groups across the three days of practice (group main effect: *p* = 0.730). Participants in both groups only demonstrated small improvements in overhand throwing performance over the course of the three days. (**B**) The absolute decrease (negative values) or increase (positive values) in endpoint error for the online, offline, and total learning effects for the tACS and SHAM groups. The online (*p* = 0.474), offline (*p* = 0.419), and total learning effects (*p* = 0.880) were similar between the tACS and the SHAM groups. The gains in online learning (reduced endpoint error) during the practice sessions were partially mitigated by offline losses (increased endpoint error) between days, which resulted in only small, non-significant improvements in total learning at the end of the three days of practice in both groups.

The 2 group (tACS, SHAM) × 3 day (1, 2, 3) mixed ANOVA revealed that the main effect for group (*p* = 0.730, η_p_^2^ = 0.006), main effect for day (*p* = 0.275, η_p_^2^ = 0.057), and day × group interaction (*p* = 0.600, η_p_^2^ = 0.023) were all non-statistically significant (Figure 3A).

The unpaired two-tailed *t*-tests revealed that the online effects (*p* = 0.474, d = 0.297), offline effects (*p* = 0.419, d = 0.336), and total learning effects (*p* = 0.880, d = 0.062) were all non-statistically significant between the tACS and SHAM groups (Figure 3B).

### 3.2. MEP Amplitude

**Figure 4:**
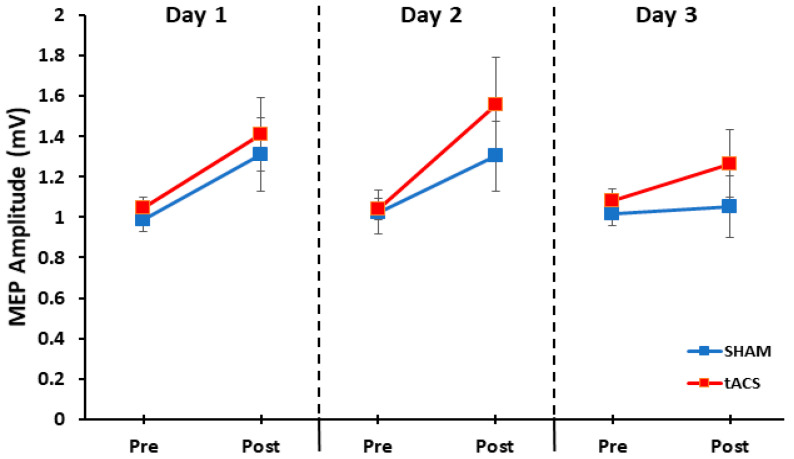
MEP amplitude for the three days in the pre-tests and post-tests in the tACS and SHAM groups. MEP amplitude was significantly increased between the pre-test and post-tests when averaged over the three days (test main effect; *p* = 0.003), but this increase was not significantly different between the tACS and SHAM groups (test × group interaction; *p* = 0.409).

The 2 group (tACS, SHAM) × 3 day (1, 2, 3) × 2 test (pre-test, post-test) three-way mixed ANOVA revealed that the main effect for group (*p* = 0.297, η_p_^2^ = 0.049) and main effect for day (*p* = 0.291, η_p_^2^ = 0.054) were both non-statistically significant. There was a significant main effect for the test (*p* = 0.003, η_p_^2^ = 0.337), which indicated that MEP amplitude was significantly greater in the post-tests compared with the pre-tests. However, the day × group interaction (*p* = 0.924, η_p_^2^ = 0.004), test × group interaction (*p* = 0.409, η_p_^2^ = 0.031), day × test interaction (*p* = 0.148, η_p_^2^ = 0.089), and day × test × group interaction (*p* = 0.813, η_p_^2^ = 0.09) were all non-statistically significant (Figure 4).

### 3.3. Futility Analyses

After the a priori sample size was attained and based on the statistical results for the primary dependent variable of endpoint error, we conducted a futility analysis [79] similar to our previous studies [44,62]. This was performed to determine if additional resources and participant recruitment were warranted. Thus, the means and SDs of the endpoint error for the tACS and SHAM groups and the effect size values reported in the statistical analyses above were used to determine the number of participants that would be needed to achieve sufficient power to find statistically significant differences in the current study. Accordingly, using the test × group interaction effect size (η_p_^2^ = 0.024) from the three-way ANOVA and the G*Power module “Test family: F tests and the statistical test ANOVA: repeated measures, within-between interaction”, it was determined that 136 participants (112 additional participants) would be needed to achieve sufficient power to find a significant interaction for endpoint error. Similar analyses using the day × group interaction effect size (η_p_^2^ = 0.023) from the two way ANOVA and the same G*Power module determined that 140 participants (116 additional participants) would be needed to achieve sufficient power to find a significant interaction for endpoint error. Finally, using the means (SHAM: −2.21 cm; tACS: −2.81 cm), SDs (SHAM: 8.87 cm; tACS: 10.67 cm), and resulting effect size (d = 0.062) from the unpaired *t*-test results for total learning and the G*Power module “Test family: *t* tests and statistical test Means: Difference between two independent means (two groups)”, it was determined that 11,196 participants (11,172 additional participants) would be needed to achieve sufficient power to find a significant interaction for endpoint error. Thus, the three different futility analyses for the three major statistical tests were in overall general agreement and revealed that it was very unlikely that the absence of significant differences between the two groups was a result of the sample size of this study. Due to the impracticality of the recruitment of these types of participants and the very low effect sizes, it was decided to stop this study for futility after the original sample size estimate was met because there were clearly no meaningful tACS treatment effects in these experimental conditions.

## 4. Discussion

The primary aim of this study was to assess the impact of tACS applied to M1 over three consecutive days of practice on the motor learning of a challenging overhand throwing task in young adults. The secondary aim was to examine the influence of tACS on M1 excitability. This study yielded five key findings: (1) the total learning effects exhibited in the overhand throwing task over the three days of practice was not significantly different between the tACS and SHAM groups; (2) both the tACS and SHAM groups displayed only small, non-significant increases in total learning effects over the three days of practice; (3) endpoint error significantly declined from the pre-tests to the post-tests during practice when averaged over the three days, but these online (within-session) effects were not statistically different between the tACS and SHAM groups; (4) the contribution of offline effects (between-sessions) to total learning was not significant and did not differ between the tACS and SHAM groups; and (5) M1 excitability as assessed by MEP amplitudes evoked during TMS testing increased from the pre-tests to the post-tests, but these increases were also not different between the tACS and SHAM groups and were not related to motor skill learning. Collectively, these findings indicate that tACS applied to M1 does not improve online, offline, or total motor learning to a greater extent than practice alone in a complex motor task.

Motor skill acquisition is a relatively transient increase in motor performance during and shortly after a practice session, whereas motor learning is a relatively more permanent improvement in movement accuracy realized through longer-time periods and extended practice [1,2]. It is thought that a minimum of 24 h [1,80] must pass from the end of practice until assessment in a retention test for changes in movement accuracy to be defined as motor learning. In addition, many of the physiological mechanisms contributing to skill acquisition and retention during the development of motor learning can be different and dependent on the characteristics of the motor task [1,80]. Although a number of brain regions contribute to motor skill acquisition and learning [2,81], decades of research have suggested that the M1 plays a major and perhaps dominant role in these processes [1,82,83,84]. Therefore, interventions that could impact physiological processes within M1 that underlie motor learning have substantial biomedical and practical significance. Accordingly, a large amount of research over the last decade and a half has indicated that tDCS delivered to M1 before or during motor practice significantly improved motor skill. However, the vast majority of this research has involved relatively simple motor tasks that were novel to the participants and practiced in a single stimulation session [11].

### 4.1. Influence of tACS on Motor Learning

Recently, a small but increasing number of studies have also applied tACS to M1 based on the idea that it may possess several unique features and advantages relative to tDCS that could be relevant to motor learning [24,29,37,38]. However, these studies have also all involved simple motor tasks performed in association with a single stimulation session [31,32,33,34]. To the best of our knowledge, the present study was the first to investigate the influence of tACS on M1 over multiple days in a complex motor task. The original central hypothesis was that the tACS application would elicit greater enhancements in motor learning in the overhand throwing task compared to SHAM stimulation. Contrary to this prediction, the total learning exhibited in the overhand throwing task from the initial pre-test block on Day 1 to the final post-test block on Day 3 was not significantly different between the tACS and SHAM groups. Specifically, both the tACS and the SHAM groups demonstrated relatively small improvements in overhand throwing accuracy, as indicated by reductions in endpoint error of only approximately −6.5% and −8.2%, respectively. This general overall pattern of results was the same if endpoint error was quantified over all the practice blocks completed each day or just in the pre- and post-test blocks. Overall, these findings are mixed relative to the most relevant and applicable previous studies on tACS and motor skill learning.

The present total learning results are not consistent with the findings of Sugata and colleagues (2018) [24], who reported that a single application of 70 Hz tACS applied with the SO-M1 electrode montage at a current strength of 1 mA elicited substantial motor skill enhancements compared to SHAM. The motor task involved a sequence comprising 12 button presses by the four fingers of the right hand, which could be viewed as a relatively simple motor task. A strength of this study was that MEG data were collected in the same study and indicated that increased beta-band power accompanied the observed behavioral improvements. Based on these strong and convincing results, this set of tACS parameters was selected for utilization in the current study. Therefore, it is somewhat difficult to reconcile the disparate findings between the two studies. However, that study did apply tACS before task practice and for only 10 min, whereas the current study used a 20-min stimulation duration and the more common tactic of stimulation application during practice. This was performed to allow as many practice trials of the overhand throwing task as possible without inducing fatigue. In addition, our previous single-day c-tDCS study [46] and a three-day M1-tDCS overhand throwing study [21] both improved motor learning with stimulation applied during practice. Overall, it seems that the differences in the complexity of the motor tasks used in the two studies are the most probable explanation for the conflicting findings. The results obtained here are also in opposition to the enhanced motor skills reported in a series of tACS studies involving index finger tracking tasks [31,32,33,34]. Similarly, Pollok and colleagues (2015) [29] found that 20 Hz tACS of M1 enhanced a four-digit motor sequence task performed with the right hand. Overall, the most relevant available studies involving M1-tACS or M1-tACS in combination with c-tACS have provided evidence that these stimulation protocols acutely increase motor skill in hand tasks. The reasons for the conflicting findings between these studies and the current study are unclear, but are likely due to the motor task involved and some differences in the tACS parameters used. In contrast, the number of stimulation sessions performed seems much less likely to have played a role, as multi-day stimulation would be predicted to be better able to display positive effects if they exist [18,19,20,21,78].

The one statistically significant behavioral finding in the current study was that endpoint error declined from the pre-tests to the post-tests during practice when averaged over the three days, which would reflect online (within-session) effects due to practice. However, this decline in endpoint error was not statistically different between the tACS and SHAM groups. Accordingly, independent of group, task practice led to a small (−5.9%) within-session decrease in endpoint error. Interestingly, these online learning effects were primarily the result of the reductions of endpoint error during practice on Day 1 (−12.7%) and Day 2 (−6.7%), as Day 3 even exhibited an increase (2.5%) between the pre- and post-tests. Although these pre- to post-test differences between days did not reach significance (day *×* test interaction: (*p* = 0.307), the pattern of results of the mean changes in endpoint error indicates that overhand throwing accuracy may have reached a short-term asymptote by the end of the second day of practice. This suggests that in this complex overhand throwing task, it may take dedicated and sustained practice over longer-term time scales of perhaps a few weeks for considerable motor skill improvements to be realized [1], at least for the current participant sample. Despite the presence of significant online effects, there was no evidence of significant offline (between-session) effects in either group. Accordingly, the tACS application did not lead to significant (positive) between-session effects, as would need to be indicated by the lower endpoint error in the pre-test block on Days 2 and 3 compared with the post-test block on Days 1 and 2, respectively. Beneficial between-session effects of tACS could have also been demonstrated if the loss of gains in endpoint performance between days was at least less compared with the SHAM group (sometimes referred to as a warm-up decrement) [1,20], but this pattern of results did not occur. Therefore, both groups displayed an equal increase in endpoint error between-sessions, as quantified by offline effects. Although this increase was not of sufficient magnitude to reach statistical significance, it was significant enough to negate much of the positive within-session online effects of practice. Consequently, the combined online and offline effects lead to the small, insignificant improvements in total learning effects exhibited by the two groups over the entirety of the three experimental sessions. Based on some prior research and assertions [1,20] as well as common real-world observations, the warm-up decrement observed in the present study was likely to have been due to the complexity of the overhand throwing task.

Taken together, the results for the online effects, offline effects, and total learning effects were unexpected and contrary to the initial hypotheses. Furthermore, the current findings are mixed relative to the most relevant and applicable previous studies from our laboratory and other research groups. More precisely, those that have involved the application of various non-invasive brain stimulation techniques to M1 or the cerebellum either in simple or complex motor tasks over multiple days. Accordingly, the rationale and experimental design were primarily based on three of our prior studies using the same overhand throwing task [21,43,46] and four studies by Reis and colleagues [18,19,20,78] that involved 3–5 days of practice of a sequential visual isometric pinch grip task (SVIPT) of the index finger and thumb. The current findings are not consistent with our previous 3-day M1-tDCS overhand throwing study, which used the same experimental protocol. Specifically, the M1-tDCS group exhibited a significant improvement in total learning compared to the SHAM group, which displayed minimal total learning. Interestingly, this significant difference was due to a combination of greater online and offline effects in the M1-tDCS group. Interestingly, the M1-tDCS group in that study achieved a 22% improvement in total learning (−22% endpoint error), which is substantially greater compared to the −8.2% in the tACS group in the current study. In another study, we applied c-tDCS during only one day of practice of the same overhand throwing task, although a retention test on the next day was used to quantify motor learning. The main findings were that the c-tDCS group enhanced total learning compared to the SHAM group, almost exclusively through online effects. However, a third study performed in our laboratory provided conflicting results from both of these prior studies. This study incorporated elements of both studies in that it involved the same 3-day experimental design and overhand throwing task but applied c-tDCS. Contrary to our expectations, the endpoint error significantly declined over the three days of practice, but the magnitude of reduction was not significant between the c-tDCS and SHAM groups. Thus, although the difference in endpoint error between the initial pre-test blocks and final post-test blocks was significant and indicated greater overall total learning effects, the between-group total learning effects were not significant, indicating that the c-tDCS application was not effective. Finally, the total learning effects that emerged independent of group were due to online effects, as the offline effects partly negated the online effects. Thus, the general results of that study show some similarities with the current study in that the stimulation group did not outperform the SHAM group. Collectively, our prior studies and the current results suggest that tACS of M1 may not be as effective as c-tDCS, and especially M1-tDCS, in improving motor learning in this difficult overhand throwing task. Ultimately, multi-day studies incorporating a complex motor task and a within-subjects design with an appropriate washout period (e.g., 1–2 months) may be needed to determine the relative effectiveness of c-tDCS, M1-tDCS, and M1-tACS on motor learning.

The absence in the literature of any other multi-day M1-tACS studies involving either a simple or a complex motor task precludes direct comparisons with the current results. However, some useful information may be able to be drawn from the mixed nature of the current findings relative to the four multi-day studies aforementioned by Reis and colleagues [18,19,20,78] that involved the SVIPT. Briefly, their initial seminal study found that M1-tDCS applied over five days during practice significantly improved total learning of the SVIPT, almost exclusively due to offline effects [20]. These results were replicated in a subsequent study [19]. In contrast, three days of c-tDCS applied during practice significantly improved SVIPT total learning, but this was primarily due to offline effects. Finally, another study reported that SVIPT total learning was significantly enhanced in a M1-SO tDCS group, a bihemispheric M1-tDCS group, and a M1-SO transcranial random noise stimulation (tRNS) group, which in all cases were due to offline effects. Collectively, the lack of M1-tACS effects in the current study is not congruent with the set of findings in all four studies, most likely due to the relative difficulty of the overhand throwing task compared to the SVIPT. Accordingly, further tACS studies comprising both simple and complex motor tasks practiced over several days are needed to determine the viability of tACS as an intervention to improve motor skills and learning.

### 4.2. Influence of tACS on M1 Excitability

tDCS applied to M1 usually increases its excitability, as indicated by enhancements in MEP amplitudes evoked by TMS for up to 90 min after stimulation [52,85], although this is not a consistent finding as many studies have not reported significant effects [86]. As previously mentioned, there are a few studies that have assessed the influence of tACS in M1 on these outcomes. The few available have also shown that tACS administered with a variety of sets of parameters can also increase M1 excitability [23,35,36], although perhaps to a lesser extent compared with tDCS. Accordingly, a unique paradigm that proved successful in two prior studies was used to assess the effects of tACS of M1 on MEP amplitude. It was initially hypothesized that the tACS group would exhibit enhanced MEPs after stimulation, whereas MEPs would be unchanged in the SHAM group. This expectation was based on not only the aforementioned tDCS and tACS results reported above by numerous research groups but also the results of our prior 3-day M1 overhand throwing study. In that study, M1-tDCS significantly increased MEP amplitudes on all three days using the identical paradigm, while MEP amplitudes in the SHAM group were unchanged. In contrast, the MEP results of the current study are exceedingly difficult to interpret and contradictory relative to our original hypothesis. Specifically, MEP amplitudes increased rather considerably following the tACS application; however, the SHAM group also displayed large MEP increases that were not too much lower relative to the tACS group. This resulted in a significant main effect of the test, as MEP amplitudes increased similarly for both groups between the pre- and post-tests, but the increase was not significantly different between the two groups.

This set of results is very counterintuitive, as SHAM stimulation does not last long enough to induce real physiological effects, and it is commonly assumed that SHAM stimulation does not induce observable placebo effects. Accordingly, the majority of tDCS and tACS studies have reported non-significant changes in MEP amplitude after stimulation. However, an often overlooked and underappreciated systematic review and meta-analysis on SHAM tDCS and related techniques reported [61] that many studies have reported increases in M1 excitability following tDCS application. Although much fewer studies were available, some data were presented that suggested similar outcomes could occur after tACS. Thus, it is not uncommon for SHAM stimulation to elicit meaningful increases in MEPs relative to baseline. Accordingly, our prior three-day c-tDCS study, which also used the same paradigm, found the same basic pattern of results as the current study. However, the magnitude of MEP increases in the SHAM group was less pronounced than those reported here (13.9% vs. 23% greater relative to baseline). In the current study, the 23% average increase in MEP amplitude in the SHAM group was not statistically different than the 35% increase in the tACS group. Thus, although MEP amplitudes increased after tACS, the same result for the SHAM group greatly complicates the interpretation of these results, especially since the 35% increase in the tACS group is in the range of those observed in tDCS studies [14]. Overall, we conservatively conclude that tACS likely had a real influence on M1 excitability. This is based on prior studies, the magnitude of the effect, and because this study was a between-subjects design. In addition, the SHAM group exhibited a very small increase in MEP amplitude on Day 3. Nevertheless, it cannot be ruled out that placebo effects or the well-known large variability and random variation common in MEP data sets were responsible for these results [14].

Finally, it was originally intended to conduct bivariate linear regression analyses to examine the relationship between changes in MEP amplitudes and changes in endpoint error, if they were to occur, as in our prior studies with the same design [21,43]. However, the outcomes deemed this inappropriate, as explained in Section 2.7. In short, this was due to the exceedingly small number of participants who exhibited an increase in both variables on the given day [21,43]. This outcome provides further support for the tDCS studies in healthy young adults that have shown no correlation between MEP amplitude increases and motor skill increases [21,43,53,58,87], which is in contrast to a few small-scale early studies in patient populations [39,40].

### 4.3. Reasons for the Failure of tACS to Improve Motor Learning and Study Limitations

The potential reasons for the lack of statistically significant results are similar to many of those that are commonly given in tDCS and tACS studies that report negative findings. In addition, most of the possible explanations are not necessarily tACS-specific but rather a reflection of the general limitations of tDCS-related techniques. Non-significant results are often unexpected due to approximately 75% of tDCS motor skill studies having demonstrated positive findings (see tables or Buch and colleagues (2017) [11]). Nonetheless, it can be seen in that review that a sizable minority of studies displayed negative findings. In addition, the possible factors responsible for the lack of significant findings, here are similar to those that our research group has reported in a number of prior studies using different forms of tDCS [43,44,45,49,63] and in a motor system fatigue study involving tACS [62]. Therefore, the most likely and relevant explanations based on tDCS studies and a few unique aspects of tACS are described only briefly here. Finally, these same possible reasons for the lack of significant findings have extensive overlap with the limitations of this study and therefore are combined within the list below. First, it could be that the tACS current level involved was not enough to elicit significant effects, and perhaps a current strength of 2 mA would have been better. Second, it has been proposed that the effects of tACS are manifested through transcutaneous stimulation of nerves on the scalp [88], which, if true, could mean that cortical structures are not modulated to a significant extent with tACS or that nerve stimulation could offset any effects. Relatedly, there could be interindividual differences [89,90] in the amount of tACS current that reaches the brain, which would have more of an influence on between-subjects designs. Third, it is also possible that tACS and other forms of non-invasive brain stimulation may not be as effective for improving complex or well-practiced motor tasks compared to simple or novel motor tasks. Similarly, there could have been ceiling effects due to the population being healthy young adults. Fourth, the tACS parameters may not have been optimal, as many different sets of parameters are possible and others have produced significant improvements in motor skill [29]. A final limitation is that the sample size of 24 was composed of 20 men and only 4 women, which could limit the generalizability of the findings. This ratio of men to women was not planned, as we simply enrolled participants as they responded to our recruitment materials as long as they met the inclusion/exclusion criteria. It was unexpected that an unbalanced number of men and women would respond to the recruitment materials, based on many of our studies in the past where relatively equal numbers of men and women volunteered in response to recruitment materials. However, we do not think the ratio of men to women significantly influenced the overall results because there is no strong evidence in the literature or in our previous tDCS and tACS studies that the percentage improvement in motor skill is different between men and women. Furthermore, the low number of women likely resulted in less variability in the data, which, if anything, should have increased the chances of finding an effect of tACS had it existed. Nonetheless, future studies that are specifically designed to directly investigate possible gender differences in the response to tACS and other forms of non-invasive stimulation are needed.

Taken together, it is most likely that the complexity of the overhand throwing task relative to the serial reaction time task (SRTT) used in many tACS [24] and tDCS studies [11,17] is likely the major reason for the lack of significant findings. Accordingly, SRTT task performance is mainly dependent on simply pushing straight down on buttons below the fingers and represents the learning of sequential motor behavior [2]. Therefore, the actual motor execution demands it possesses are very small [2] and much lower than in an overhand throwing task. Accordingly, primarily isometric finger sequence tasks performed unilaterally with the fingers of one hand do not possess the movement complexities of overhand throwing summarized above (Section 2.5). Nonetheless, future studies are needed to systematically isolate and address each of these individual possibilities relative to the heterogeneity of the influence of tACS on motor skill learning.

### 4.4. Practical Applications

One of the reasons that non-invasive brain stimulation methods such as tACS and tDCS have been studied so extensively in recent years is that they are easily able to be put into practical use. Although the current study involving a very difficult motor task in an active young population found no effect of concurrent tACS application on motor learning, many other studies using simple motor tasks have shown positive effects [24,31,32,33,34]. In addition, it is more likely that the technique could be efficacious in older or patient populations. Specifically, tACS and related methods could be used to address the various motor performance-related impairments (e.g., fine motor control, gait function, movement speed, and fatigue) such as in diseases such as multiple sclerosis [91,92,93] and Parkinson’s disease [44,94,95]. Therefore, there are several practical applications that are relevant to the current results. First, tACS could be applied before motor task practice or the execution of an important task associated with an activity of daily living or in the workplace. This could be especially valuable if it was not possible to apply the stimulation during the task or if the task involved motor sequence tasks of the hand, such as keyboarding [24]. Second, tACS likely has greater practical applications in the above scenarios in older adults or in certain movement disorder populations where there is more room for improvement in motor skills due to much less of a ceiling effect than is present in young adults. Furthermore, the affordability and ease of use make tACS devices easily able to be used in the home by a patient themselves or by a caregiver. Third, there are practical research implications to this study, as it has been shown that tDCS and tACS devices can be successfully used by both individuals with Parkinson’s disease and multiple sclerosis in research studies with remote online supervision and instruction by investigators [96,97,98,99]. In summary, future research should be directed at determining which specific motor tasks can be improved by tACS in various populations so that practical strategies for implementation can be devised and implemented successfully.

## 5. Conclusions

The current study was the first to investigate the influence of tACS of M1 applied over multiple days on motor learning in a complex motor task. Overall, the main findings were that the online, offline, and total learning effects of a complex motor task were not significantly different between the tACS and SHAM groups. Therefore, the results were in contrast to most of the available M1-tACS studies that have investigated simple motor tasks. Further research is needed to understand and determine the ability of tACS in M1 to improve motor skills and learning in different motor tasks. There is significant room in these areas, as much less study has been performed on tACS in these areas compared to other non-invasive brain stimulation methods. Future research should incorporate concurrent physiological and behavioral measurements to characterize the mechanisms underlying any observed effects of tACS on motor skill and learning.

## Data Availability

The data presented in this study are available on request from the corresponding author.
